# Supplementary material for: DMRforPairs: identifying Differentially Methylated Regions between unique samples using array based methylation profiles
Source: BMC Bioinformatics. 2014 May 15;15:141. doi: 10.1186/1471-2105-15-141 (PMC4046028; doi:10.1186/1471-2105-15-141)
Supplement: Additional file 2 — DMRforPairs output for the comparison of A431-MCF7 and NA17018-NA17105. Please start from the HTML files in each folder. Available via the BMC Bioinformatics website. [file 1471-2105-15-141-S2.zip › 1394847754114233_MOESM2_ESM/A431_MCF7/figures/3369.pdf]

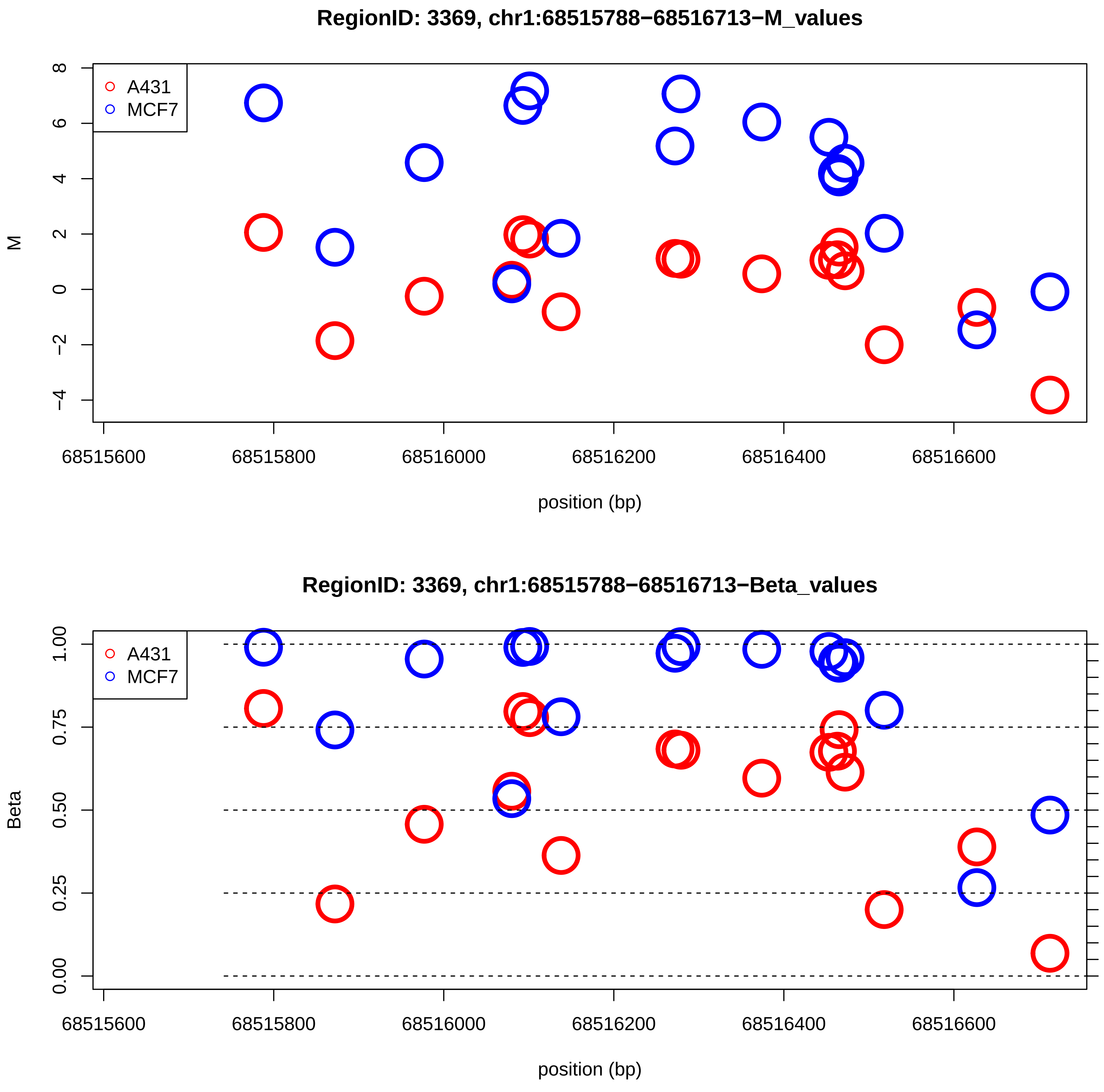
RegionID: 3369, chr1:68515788−68516713−M_values
A431
MCF7
M
8
6
4
2
0
−2
−4
68515600
68515800
68516000
68516200
68516400
68516600
position (bp)
RegionID: 3369, chr1:68515788−68516713−Beta_values
A431
MCF7
Beta
1.00
0.75
0.50
0.25
0.00
68515600
68515800
68516000
68516200
68516400
68516600
position (bp)

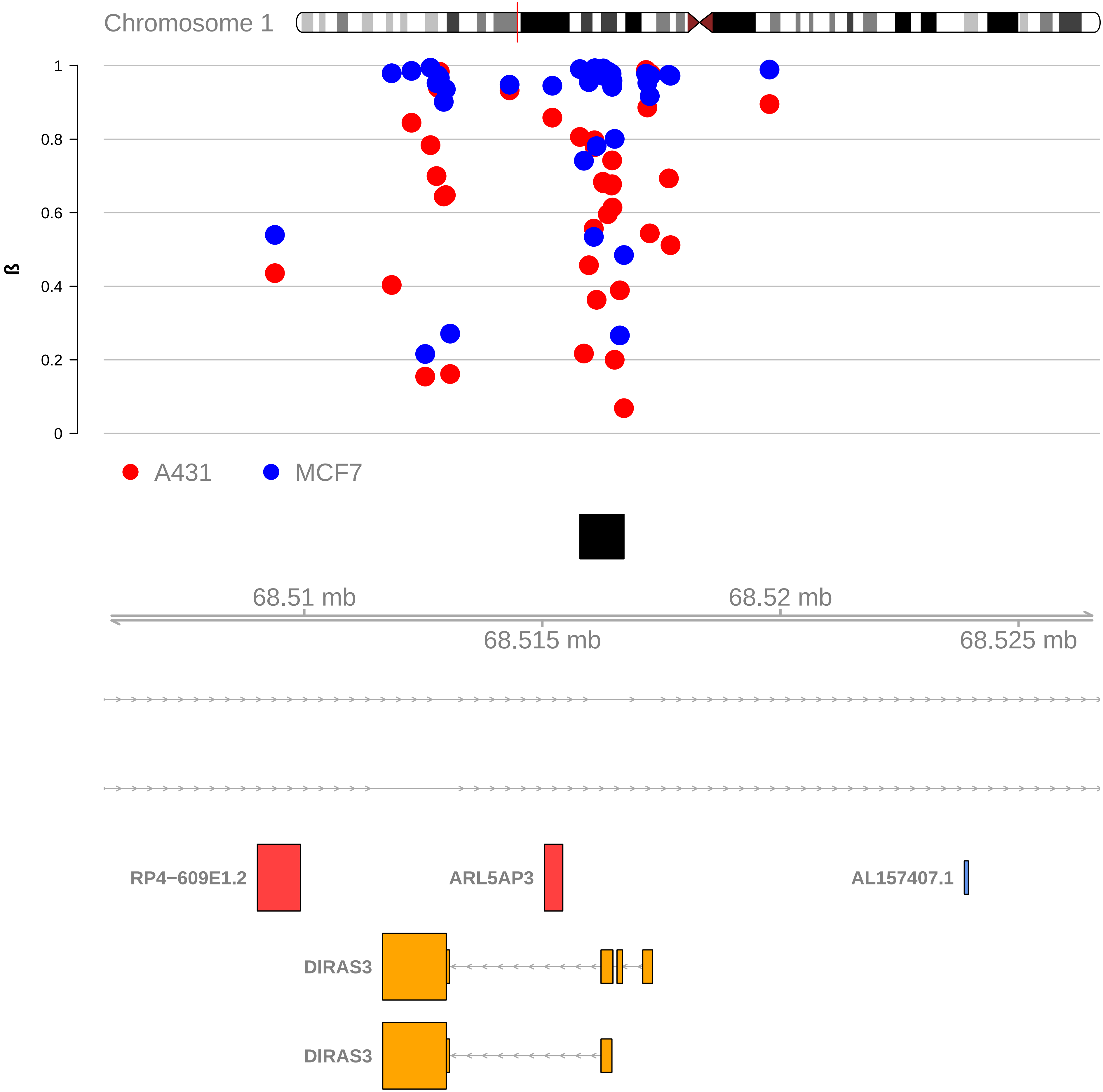

Chromosome 1
1
0.8
0.6
0.4
0.2
0
ß
A431
MCF7
68.51 mb
68.52 mb
68.515 mb
68.525 mb
RP4−609E1.2
ARL5AP3
AL157407.1
DIRAS3
DIRAS3
